# An Adaptive Learning Rate for RBFNN Using Time-Domain Feedback Analysis

**DOI:** 10.1155/2014/850189

**Published:** 2014-03-20

**Authors:** Syed Saad Azhar Ali, Muhammad Moinuddin, Kamran Raza, Syed Hasan Adil

**Affiliations:** ^1^Department of Electrical and Electronic Engineering, Universiti Teknologi Petronas, Bandar Seri Iskandar, 31750 Tronoh, Perak, Malaysia; ^2^Faculty of Engineering, Sciences and Technology, Iqra University, Defence View, Karachi 75500, Pakistan

## Abstract

Radial basis function neural networks are used in a variety of applications such as pattern recognition, nonlinear identification, control and time series prediction. In this paper, the learning algorithm of radial basis function neural networks is analyzed in a feedback structure. The robustness of the learning algorithm is discussed in the presence of uncertainties that might be due to noisy perturbations at the input or to modeling mismatch. An intelligent adaptation rule is developed for the learning rate of RBFNN which gives faster convergence via an estimate of error energy while giving guarantee to the *l*_2_ stability governed by the upper bounding via small gain theorem. Simulation results are presented to support our theoretical development.

## 1. Introduction

Neural Networks have recently been used in almost every field of science. Radial basis function neural networks (RBFNN) are single-layered feedforward networks with universal approximation capabilities, in addition to more efficient learning than the famous multilayered feedforward neural networks (MFNN) [1]. RBFNN has been used in a wide variety of applications such as in [2–9].

RBFNN are generally trained using supervised learning. During training, a recursive update procedure is used to estimate the weights of the RBFNN that best fit the given data [1]. The recursive procedure often requires selecting a suitable adaptation gain called learning rate. The learning rate should be within an optimum range. It should neither be too large which would drive the algorithm unstable, nor too small that it slows down the training. In general practice, trial-and-error experiences are used to select a suitable learning rate for training phase.

Due to inherent nonlinearity in the structure of neural networks, its convergence analysis becomes complicated. A very limited work has been done in this context. In [8–10], the authors have presented a robustness analysis for the perceptron neural network. The formulation in [8–10] emphasizes an intrinsic feedback structure for most adaptive algorithms and it relies on tools from system theory, control, and signal processing such as state-space description, feedback analysis, small gain theorem, *H* ^*∞*^ design, and lossless systems. The feedback configuration is provoked via energy arguments and is shown to consist of two major blocks: a time-variant* lossless* (i.e., energy preserving) feedforward path and a time-variant feedback path.

More recently, in [11], convergence analysis of RBFNN is carried out and optimal adaptation for its learning rate is derived using the deterministic feedback analysis aided with small gain theorem. But the work does not include any adaptive mechanism for the learning rate in true sense. In contrast, in this work, we present an intelligent adaptation rule for the learning rate of RBFNN which gives faster convergence via an estimate of error energy while giving guarantee to the *l*_2_ stability governed by the upper bounding via small gain theorem. Moreover, unlike the work of [11], we avoid mean value theorem, thanks to the RBFNN structure which allows us to separate the nonlinearity with its weights as opposed to the perceptron structure where it is not possible to separate them. This in turn helps us to avoid mean value theorem by using the relation of a priori estimation error. Another distinguished and good feature of our work in contrast to the work in [11] is that it does not require the calculation of the derivative of the radial basis function for the learning rate adaptation.

The paper is organized as follows. Following the introduction in Section 1, we present overview of RBFNN in Section 2.  Section 3 develops a deterministic framework for the robustness analysis of RBFNN. The feedback structure for lossless mapping is provided in Section 4 and as a result a stability bound is derived in Section 5. In Section 6, an intelligent adaptive rule is presented for the learning rate of RBFNN. Simulation results are presented in Section 7 to validate our theoretical findings. Finally, the concluding remarks are given in Section 8.

## 2. Radial Basis Functions Neural Networks

**Figure 1:**
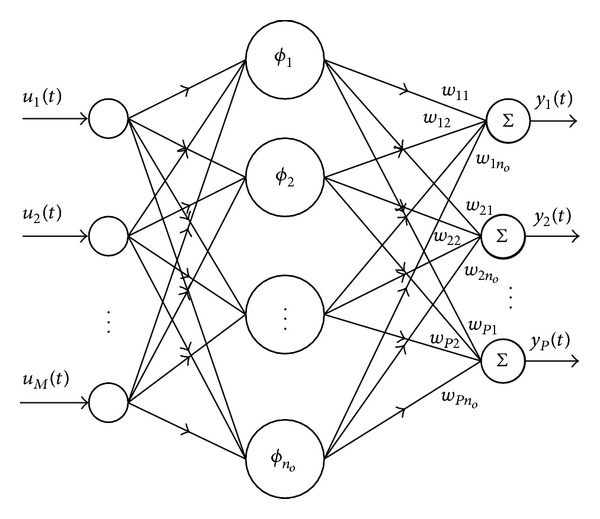
A MIMO RBF neural network.

RBFNN is a type of feedforward neural network. They are used in a wide variety of contexts such as function approximation, pattern recognition, and time series prediction. Networks of this type have the universal approximation property [1]. In these networks the learning involves only one layer with lesser computations. A multi-input multioutput RBFNN is shown in Figure 1. The RBFNN consists of an input node *u*(*t*), a hidden layer with *n*_*o*_ neurons, and an output node *y*(*t*). Each of the input nodes is connected to all the nodes or neurons in the hidden layer through unity weights (direct connections). While each of the hidden layer nodes is connected to the output node through some weights, for example, the *i*th output node is connected with all the hidden layer nodes by *W*(*t*) = [*w*_1_(*t*),…, *w*_*n*_*o*__(*t*)], each neuron finds the distance, normally applying Euclidean norm, between the input and its center and passes the resulting scalar through a nonlinearity. So the output of the *i*th hidden neuron is given by *ϕ*_*i*_(||*u*(*t*) − *c*_*i*_||), where *c*_*i*_ is the center of the *i*th hidden layer node, *i* = 1,2,…, *n*_*o*_, and *ϕ*_*i*_(·) is the nonlinear basis function. Normally this function is taken as a Gaussian function of width *β*, which dictates the effective range of input passing through the basis function. The output *y*_*m*_(*t*) is a weighted sum of the outputs of the hidden layer, given by

(1) $$\begin{array}{c} y_{m}\left(t\right)=\Phi \left(t\right)W\left(t\right),\\ y_{m}\left(t\right)=\sum_{i=1}^{n_{o}}\phi_{i}\left(||u\left(t\right)-c_{i}||\right)w_{i}\left(t\right), \end{array}$$

where the basis functions and weight vector are defined as

(2) $$\begin{array}{c} \Phi \left(t\right)=\left[\begin{array}{ccc} \phi_{1}\left(u\left(t\right)\right)\;\;\phi_{2}\left(u\left(t\right)\right) & \cdots & \phi_{n_{o}}\left(u\left(t\right)\right) \end{array}\right], \end{array}$$

and the Gaussian basis function is

(3) $$\begin{array}{c} \phi_{i}\left(u\left(t\right)\right)=\exp\left(-\frac{{||u\left(t\right)-C_{i}||}^{2}}{\beta^{2}}\right). \end{array}$$

Consider a collection of input vectors {*u*(*t*)} with the corresponding desired output vectors {*y*(*t*)}. We also take into account noisy perturbations *v*(*t*) in the desired signal. These perturbations can be due to model mismatch or to measurement noise. Assuming there exists an optimal weight vector *W*_*o*_ such that

(4) $$\begin{array}{c} y\left(t\right)=\Phi \left(t\right)W_{o}+v\left(t\right). \end{array}$$

The RBFNN is presented with the given input-output data {*u*(*t*), *y*(*t*)}. The objective is to estimate the unknown optimal weight *W*_*o*_. Now, starting with an initial guess *W*_0_, the weights are updated recursively based on the LMS principle as

(5) $$\begin{array}{c} W\left(t+1\right)=W\left(t\right)+\alpha \left(t\right)e\left(t\right)\Phi^{T}\left(t\right), \end{array}$$

where *α*(*t*) is the learning and the error *e*(*t*) is defined as

(6) $$\begin{array}{c} e\left(t\right)=y\left(t\right)-y_{m}\left(t\right),\\ e\left(t\right)=\Phi \left(t\right)W_{o}-\Phi \left(t\right)W\left(t\right)+v\left(t\right). \end{array}$$

We define* a priori* and* a posteriori* error quantities as

(7) $$\begin{array}{c} e_{a}\left(t\right)=\Phi \left(t\right)\tilde{W}\left(t\right),\\ e_{p}\left(t\right)=\Phi \left(t\right)\tilde{W}\left(t+1\right), \end{array}$$

where $\tilde{W}(t)$ is the weight error vector symbolizing the difference between the optimal weight and its estimate as $\tilde{W}(t)=W_{o}-W(t)$. Thus, we can rewrite the *e*_*a*_(*t*) as

(8) ea(t)=Φ(t)Wo−Φ(t)W(t)

(9) =Φ(t)Wo−ym(t),

(10) ep(t)=Φ(t)[W~(t)−α(t)Φ(t)Te(t)]=ea(t)−α(t)||Φ(t)||2e(t).

Consequently, the weight error update equation satisfies the following recursion:

(11) $$\begin{array}{c} \tilde{W}\left(t+1\right)=\tilde{W}\left(t\right)-\alpha \left(t\right)e\left(t\right)\Phi^{T}\left(t\right). \end{array}$$

## 3. A Deterministic Framework for the Robustness of RBFNN

Robustness of an algorithm is defined as the consistency in its estimation error with the disturbances in the sense that a minor increase in disturbances would lead to a smaller increase in its estimation error irrespective of the disturbances nature. In order to study the robustness of RBFNN, we employ a pure deterministic framework without assuming any prior knowledge of signal or noise statistics as was used in [8, 9]. This is especially useful in situations where prior statistical information is missing. The robust design would guarantee a desired level of robustness independent of the noise statistics. In a broad sense, robustness would imply that the ratio of an estimation error energy to the noise or disturbance energy will be guaranteed to be upper bounded by a positive constant:

(12) $$\begin{array}{c} \frac{\text{estimation}\;\;\text{error}\;\;\text{energy}}{\text{disturbance}\;\;\text{energy}}\leq 1. \end{array}$$

Thus, the ratio in (12) gives the assurance that the resulting estimation error energy will be upper bounded by the disturbance energy, regardless of the nature and statistics of noise.

Next, we will develop a lossless mapping between the estimation errors while adapting the weights from the *t*th time instant to the (*t* + 1)th time instant. A lossless mapping is the one that transforms *x* to *y* as *y* = *T*[*x*] in such a way that we have ||*T*[*x*]||^2^ ≤ ||*x*||^2^ for all *x*; that is, the output energy does not exceed the input energy. To set up the stage for the analysis, we define the disturbance error $\tilde{v}(t)$ as

(13) $$\begin{array}{c} \tilde{v}\left(t\right)=e\left(t\right)-e_{a}\left(t\right). \end{array}$$

Now, by using the above definition and definitions of estimation errors, we evaluate the energies of both sides of the weight error recursion (11) as follows:

(14) ||W~(t+1)||2=||W~(t)||2−2α(t)e(t)Φ(t)W~(t) +α(t)2e2(t)||Φ(t)||2,=||W~(t)||2−2α(t)ea2(t)−2α(t)ea(t)v~(t) +α(t)2||Φ(t)||2ea2(t) +2α(t)2||Φ(t)||2ea(t)v~(t) +α(t)2||Φ(t)||2v~2(t).  

By rearranging the relevant terms, we finally arrive at

(15) ||W~(t+1)||2+2α(t)ea2(t)−α(t)2μ(t)ea2(t) =||W~(t)||2−2α(t)ea(t)v~(t)+2α(t)2μ(t)ea(t)v~(t)  +α(t)2μ(t)v~2(t),

where we have introduced a new parameter *μ*(*t*) defined as

(16) $$\begin{array}{c} \mu \left(t\right)=\frac{1}{{||\Phi \left(t\right)||}^{2}}. \end{array}$$

Thus, it can be easily seen from the mapping (15) that the following three different scenarios exist depending upon the value of learning rate:

(17) $$\begin{array}{c} \frac{{||\tilde{W}\left(t+1\right)||}^{2}+\alpha \left(t\right)e_{a}^{2}\left(t\right)}{{||\tilde{W}\left(t\right)||}^{2}+\alpha \left(t\right){\tilde{v}}^{2}\left(t\right)}\{\begin{array}{cc} \leq 1, & \text{for}\;\;0<\alpha \left(t\right)<\mu \left(t\right)\\ =1, & \text{for}\;\;\alpha \left(t\right)=\mu \left(t\right),\\ \geq 1, & \text{for}\;\;\alpha \left(t\right)>\mu \left(t\right). \end{array} \end{array}$$

The first two inequalities in the statement of (3) ascertain that if the learning rate is chosen such that *α*(*t*) ≤ *μ*(*t*), then the mapping from signals $\left\{\tilde{W}(t),\sqrt{\mu (t)}e_{p}(t)\right\}$ to the singals $\left\{\tilde{W}(t+1),\sqrt{\mu (t)}e_{a}(t)\right\}$ is a lossless or contractive mapping. Therefore, a local energy bound is deduced that highlights a robustness property of the update recursion. The energy bound depicts that no matter what the value of the noise component $\tilde{v}(t)$ is, and no matter how far the estimate *W*(*t*) is from the optimal *W*_*o*_, the sum of energies ${||\tilde{W}(t+1)||}^{2}+\alpha (t)e_{a}^{2}(t)$ will always be smaller than or equal to the sum of energies ${||\tilde{W}(t)||}^{2}+\alpha (t){\tilde{v}}^{2}(t)$. Since this contractive property holds for each *t*th instant, it should also hold globally over any interval. In fact, selecting *α*(*t*) < *μ*(*t*) over the interval 0 ≤ *t* ≤ *N*, it follows that

(18) $$\begin{array}{c} {||\tilde{W}\left(N\right)||}^{2}+\sum_{t=0}^{N}\alpha \left(t\right)e_{a}^{2}\left(t\right)\leq {||\tilde{W}\left(0\right)||}^{2}+\sum_{t=0}^{N}\alpha \left(t\right){\tilde{v}}^{2}\left(t\right). \end{array}$$

## 4. Feedback Structure for Lossless Mapping

In this section, a feedback structure is established that explains a lossless mapping between estimation errors *e*_*a*_(*t*) and *e*_*p*_(*t*). To do so, we first reformulate the a posteriori error defined in (10) in terms of parameter *μ*(*t*) as follows:

(19) $$\begin{array}{c} e_{p}\left(t\right)=e_{a}\left(t\right)-\alpha \left(t\right){||\Phi \left(t\right)||}^{2}e\left(t\right),\\ \alpha \left(t\right)e\left(t\right)=\mu \left(t\right)\left(e_{a}\left(t\right)-e_{p}\left(t\right)\right). \end{array}$$

Hence, the weight error recursion in (11) will take the following form:

(20) $$\begin{array}{c} \tilde{W}\left(t\right)=\tilde{W}\left(t\right)-\mu \left(t\right)\Phi^{T}\left(t\right)\left(e_{a}\left(t\right)-e_{p}\left(t\right)\right). \end{array}$$

Thus, the evaluation of energies of the both sides of the above equation leads to a similar form as (3) with equality showing a lossless mapping between the estimation errors and it is found to be

(21) $$\begin{array}{c} \frac{{||\tilde{W}\left(t+1\right)||}^{2}+\mu \left(t\right)e_{a}^{2}\left(t\right)}{{||\tilde{W}\left(t\right)||}^{2}+\mu \left(t\right)e_{p}^{2}\left(t\right)}=1, \end{array}$$

which holds for all possible choices of the learning rate. This implies that the mapping ${\bar{T}}_{i}$ from the signals $\left\{\tilde{W}(t),\sqrt{\mu (t)}e_{p}(t)\right\}$ to the signals $\left\{\tilde{W}(t+1),\sqrt{\mu (t)}e_{a}(t)\right\}$ is lossless.

Next, by employing the relations (8) and (6), (19) can be set up as

(22) $$\begin{array}{c} e_{p}\left(t\right)=e_{a}\left(t\right)-\frac{\alpha \left(t\right)}{\mu \left(t\right)}\left(e_{a}\left(t\right)+v\left(t\right)\right),\\ e_{p}\left(t\right)=\left[1-\frac{\alpha \left(t\right)}{\mu \left(t\right)}\right]e_{a}\left(t\right)-\frac{\alpha \left(t\right)}{\mu \left(t\right)}v\left(t\right),\\ -\sqrt{\mu \left(t\right)}e_{p}\left(t\right)=\frac{\alpha \left(t\right)}{\sqrt{\mu \left(t\right)}}v\left(t\right)-\left[1-\frac{\alpha \left(t\right)}{\mu \left(t\right)}\right]\sqrt{\mu \left(t\right)}e_{a}\left(t\right). \end{array}$$

**Figure 2:**
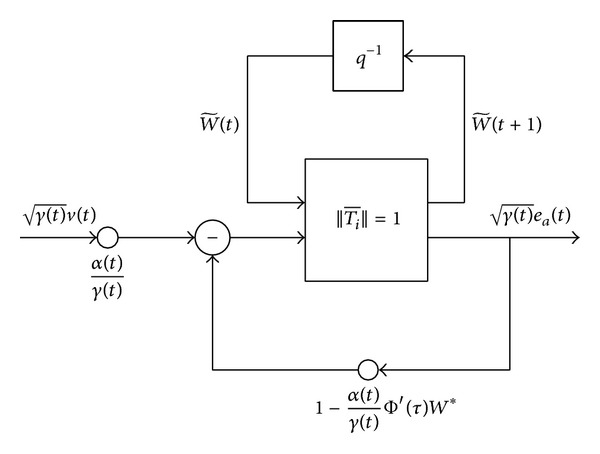
A lossless mapping in a feedback structure for RBFNN learning algorithm.

This relation shows that the overall mapping from the* original* (weighted) disturbances $\sqrt{\mu (t)}v(t)$ to the resulting* a priori* (weighted) estimation errors $\sqrt{\mu (t)}e_{a}(t)$ can be expressed in terms of a feedback structure, as shown in Figure 2.

## 5. Stability Bound via Small Gain Theorem

The stability of the structures of the form (22) can be studied via well-known tools such as the small gain theorem [12]. Thus, conditions on the learning rate *α*(*t*) will be derived in order to guarantee a robust training algorithm, as well as faster convergence speeds.

This will be achieved by establishing conditions under which the feedback configuration is *l*_2_ stable in the sense that it should map a finite-energy input noise sequence (which includes the noiseless case a special case) $\left\{\sqrt{\mu (t)}v(t)\right\}$ to a finite-energy* a priori* error sequence $\left\{\sqrt{\mu (t)}e_{a}(t)\right\}$.

The small gain theorem for our scenario can be stated as

(23) $$\begin{array}{c} \Delta \left(N\right)=\underset{0\leq t\leq N}{\max}\left|1-\frac{\alpha \left(t\right)}{\mu \left(t\right)}\right|. \end{array}$$

According to the above definition, Δ(*N*) is the maximum absolute gain of the feedback loop over the interval 0 ≤ *t* ≤ *N*.

The small gain theorem states that the *l*_2_ stability of a feedback configuration such as the configuration in Figure 2 as special case requires that the product of norms of the feedforward and feedback maps be strictly bounded by one [8, 9, 12]. In our case, the norm of the feedforward map is equal to one (since it is lossless) while the norm of the feedback map is defined in (23) as Δ(*N*). Hence, the condition Δ(*N*) < 1 guarantees an overall contractive map. Therefore, for Δ(*N*) < 1 to hold, we need to choose the learning rate such that, for all *t*,

(24) $$\begin{array}{c} 0<\alpha \left(t\right)<2\mu \left(t\right)=\frac{2}{{||\Phi \left(t\right)||}^{2}}. \end{array}$$

## 6. Designing Adaptive Learning Rate

In this section, we propose an adaptive mechanism to update the learning rate *α*(*t*) such that it gives faster convergence as well as guaranteeing the *l*_2_ stability discussed in the previous section. For this, we propose an adaptive mechanism similar to the one in [13] according to which the learning rate should be adapted via an estimate of error correlation. In addition, we upper-bounded the maximum value of the learning rate to assure its *l*_2_ stability by employing the stability bound derived in (24). To do so, we propose the following adaptive rule [13]:

(25) $$\begin{array}{c} \alpha \left(t+1\right)=\lambda \eta \left(t\right)+\gamma e^{2}\left(t\right),\;\left(0<\lambda <1,\gamma >0\right), \end{array}$$

(26) $$\begin{array}{c} \eta \left(t+1\right)=\{\begin{array}{cc} \eta_{\max}, & \text{if}\;\;\alpha \left(t+1\right)>\eta_{\max},\\ \alpha \left(t+1\right), & \text{otherwise}, \end{array} \end{array}$$

where the parameter *η*_max⁡_ is so chosen that it ensures *l*_2_ stability given in (23). Thus, *η*_max⁡_ is given by

(27) $$\begin{array}{c} \eta_{\max}=\frac{2}{{||\Phi \left(t\right)||}^{2}}, \end{array}$$

where the parameter *λ* is a positive quantity showing its dependency on its own past value and lies in the range [0,1] (usually we choose a value closer to 1, e.g., 0.97) while the constant *γ* is a very small number. The adaptation rule given by (25) and (26) suggests that the learning rate is large in the initial stage of adaptation due to larger error correlation and it decreases near steady state as the error correlation of the algorithm also decreases once the algorithm approaches the steady state. Thus, by adjusting the learning rate online according to the rule given in (25), it will give faster convergence as it allows faster adaptation of *α*(*t*) via an estimate of error energy due to the term *γe* ^2^(*t*). On the other hand, the adaptation rule in (24) will guarantee the stability of the feedback structure due to upper bounding via the stability limit in (24), that is, 2/||Φ(*t*)||^2^. Thus, it promises both faster convergence and a stable response.

## 7. Simulation Results

The proposed adaptive learning rate is verified using various simulations for nonlinear identification and tracking control. In all the cases the simulation is first performed for fixed learning rates. The fixed learning rates are set after several trials. However, these trials are not required when using the proposed adaptive learning rate given by (24), (25), and (26). A comparison for different fixed learning rates and adaptive learning rate is shown for each example along with identification/tracking and learning rate trends.

### 7.1. Identification of Nonlinear Control Valve

In this simulation example, the proposed adaptive learning rate is used in the identification of a model that describes a valve for control of fluid flow described in [14] as

(28) $$\begin{array}{c} y\left(t\right)=\frac{u\left(t\right)}{\sqrt{0.10+0.90u^{2}\left(t\right)}}. \end{array}$$

**Figure 3:**
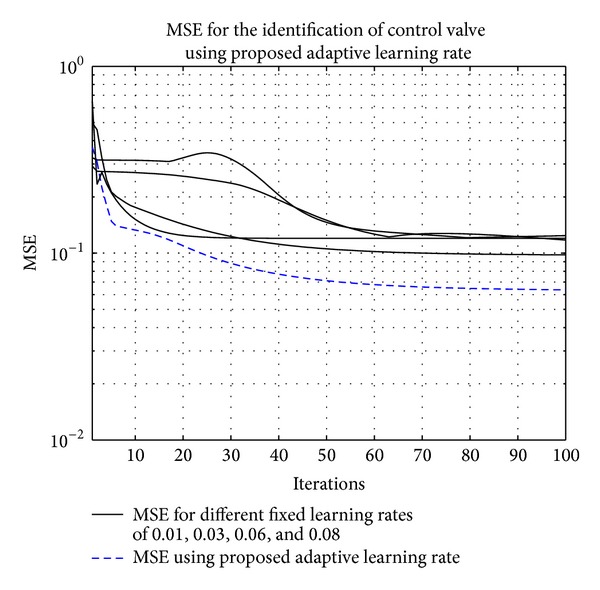
MSE for the identification of control valve using fixed and adaptive learning rates. The fixed learning rates are 0.01, 0.03, 0.06, and 0.08.

The model is identified using an RBFNN with 5 centers spaced at 0.5. The width of the center is set to 0.6. An output additive noise of 30 dB SNR is considered in this example. Learning rates of 0.01, 0.03, 0.06, and 0.08 are used for fixed learning rate case. The algorithm became unstable and values are near or greater than 0.08. After the simulations, mean square errors (MSE) for fixed and adaptive learning rates are shown in Figure 3. The lowest MSE achieved using adaptive learning rate shows the performance of the proposed approach.

**Figure 4:**
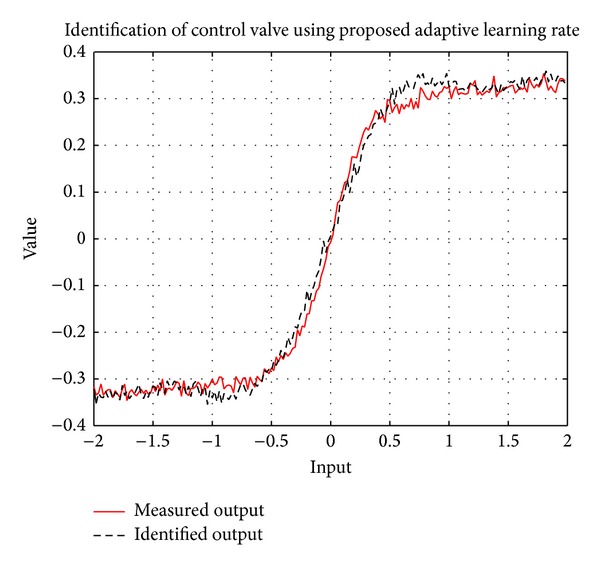
Actual and identified control valve using the proposed adaptive learning rate.

**Figure 5:**
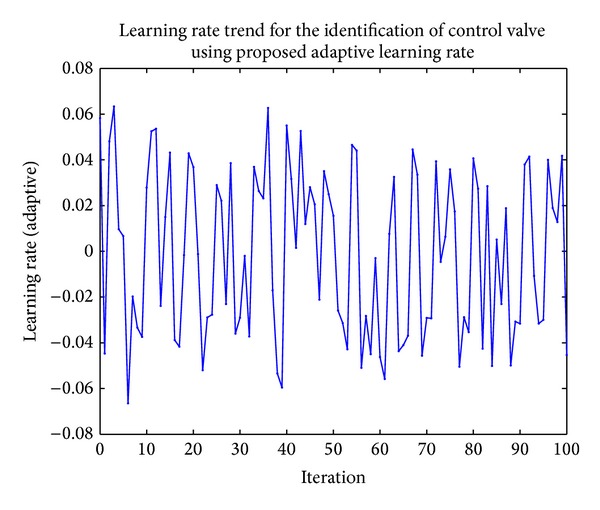
Learning rate trend for the identification of control valve.

Actual and identified control valve using the proposed approach are shown in Figure 4. The learning rate trend can be seen in Figure 5.

### 7.2. Identification of Nonlinearity in Hammerstein Model

The proposed bound on the adaptive learning rate is used in the identification of the static nonlinearity in nonlinear Hammerstein model defined in [15]. The Hammerstein model has been identified using RBFNN in [16]. The Hammerstein model used for simulation represents a nonlinear heat exchanger in cascade with linear dynamics. The static nonlinearity and linear dynamics are given by [15]

**Figure 6:**
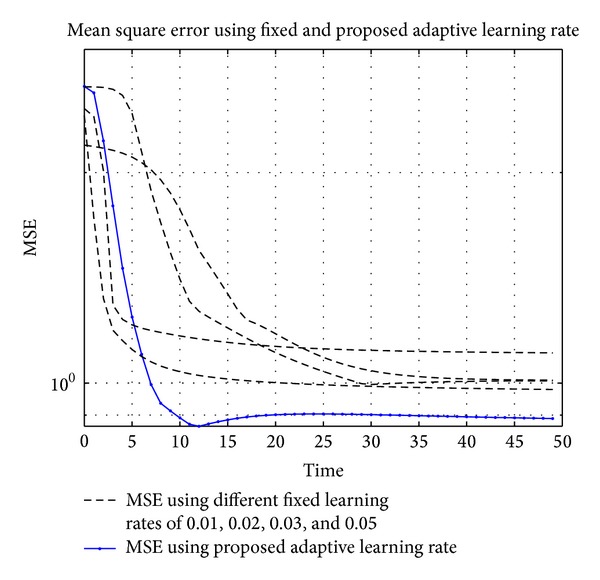
MSE for the identification of heat exchanger in Hammerstein model using fixed and adaptive learning rates. The fixed learning rates are 0.01, 0.02, 0.03, and 0.05.

**Figure 7:**
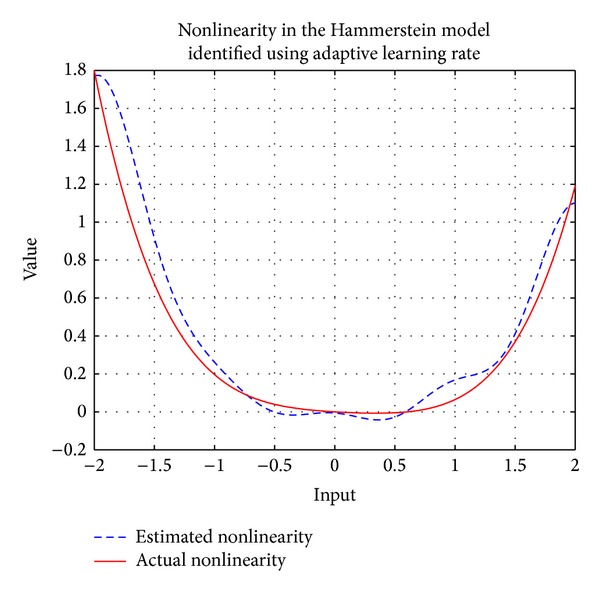
Actual and identified heat exchanger in Hammerstein model using the proposed adaptive learning rate.

(29) x(t)=−31.549u(t)+41.732u2(t)−24.201u3(t)   +68.634u4(t),y(t)=0.4y(t−1)+0.35y(t−2)+0.15x(t)+v(t),

where *u*(*t*) is the input to the system, *x*(*t*) is the intermediate variable, *y*(*t*) is the system output, and *v*(*t*) is additive noise at the output. Actual and identified heat exchangers using the proposed approach are shown in Figure 7. The nonlinearity in the Hammerstein model is identified online using RBFNN with 9 neurons and a width of 0.5. An output additive noise of 30 dB SNR is considered in this example. Learning rates of 0.01, 0.02, 0.03, and 0.05 are used for fixed learning rate case. The algorithm became unstable at values near or greater than 0.07. After the simulations mean square errors (MSE) for fixed and adaptive learning rates are shown in Figure 6, the lowest MSE achieved using adaptive learning rate shows the performance of the proposed approach.

**Figure 8:**
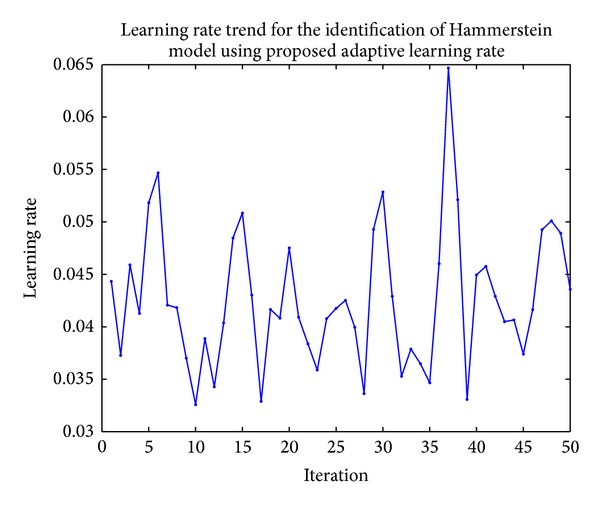
Learning rate trend for the identification of heat exchanger in Hammerstein model.

The learning rate trend can be seen in Figure 8.

### 7.3. Adaptive Inverse Control Using RBFNN

In this simulation example an adaptive control technique, namely, adaptive inverse control (AIC) is considered [17]. This technique is based on identifying the plant and its inverse. This technique was introduced for stable, minimum phase linear systems; however, with appropriate modification it can also be used for nonminimum phase and nonlinear systems [17].

Consider a nonminimum phase plant with a transfer function:

(30) $$\begin{array}{c} G\left(s\right)=\frac{s\left(s+1\right)}{s^{2}+0.45s+0.1}. \end{array}$$

AIC based on RBFNN is used for tracking control of the given plant. Therefore, the plant is identified online using RBFNN with 5 centers and width of 1. The weights are initialized with random numbers. These initial weights are kept the same for all different cases of learning rates. The response to a square wave with output additive noise is measured and compared with the RBFNN output. The mismatch signal is used as error signal in weight update (5).

**Figure 9:**
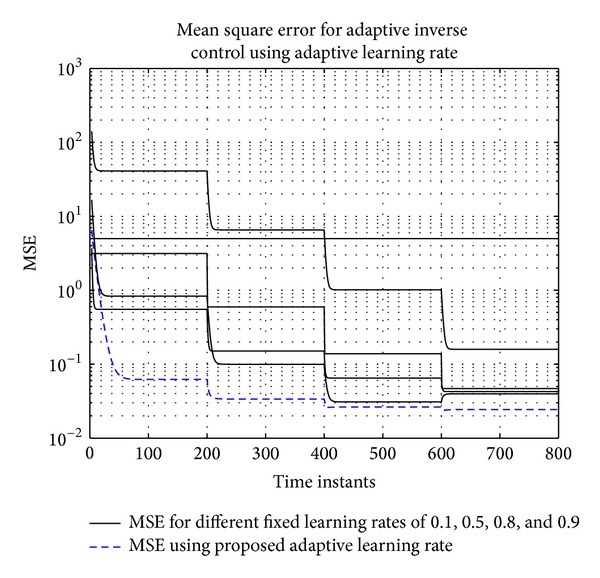
MSE for tracking of nonminimum phase plant in AIC using fixed and adaptive learning rates. The fixed learning rates are 0.1, 0.5, 0.8, and 0.9.

For fixed learning rate case, learning rates of 0.1, 0.5, 0.8, and 0.9 are used. The algorithm became unstable at learning rate of 1. MSE for different learning rates and proposed adaptive learning rate are shown in Figure 9. It is observed that the MSE converges faster with higher learning rates. On the other hand, using the proposed adaptive learning rate MSE converges to smaller values than any of the fixed learning rate cases.

**Figure 10:**
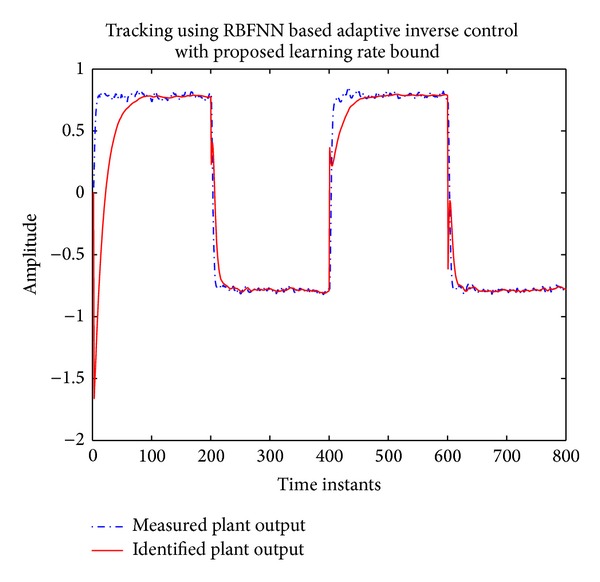
Tracking using RBFNN based AIC with Proposed Learning Rate Bound.

**Figure 11:**
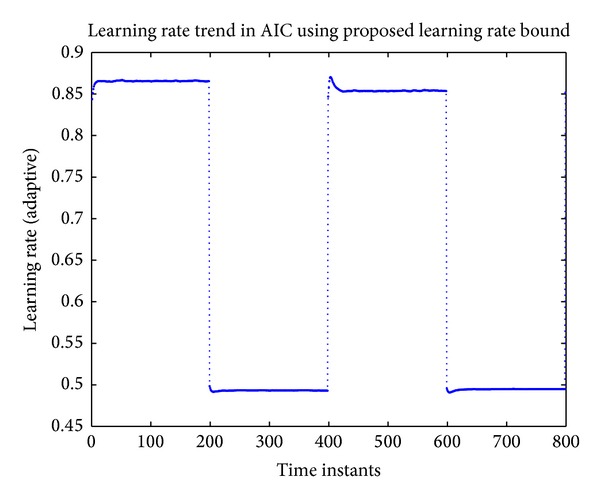
Learning rate trend for the tracking using RBFNN based AIC.

The tracking of actual and identified nonminimum phase plant using the proposed approach is shown in Figure 10. The learning rate trend can be seen in Figure 11.

### 7.4. Internal Model Control of MIMO System Using RBFNN Based U-Model

In this simulation example internal model control (IMC) [18] is applied for the tracking control of a 2-input 2-output system. The plant is modelled by RBFNN based U-model. The details of the RBFNN based U-model are presented in [19, 20].

The 2-input 2-output system is given by

(31) y1(t)=0.21y1(t−1)−0.12y2(t−2) +0.3y1(t−1)u2(t−1)−1.6u2(t−1)+v1(t),y2(t)=0.25y2(t−1)−0.1y1(t−2) −0.2y2(t−1)u1(t−1)+1.2u1(t−1)+v2(t),

where *y*_*s*_ are the output, *u*_*s*_ are the input, and *v*_*s*_ are output additive noise.

IMC based on U-model is used for tracking control of the MIMO system. Therefore, the plant is identified online using 2-input 2-output RBFNN based U-model [19] with 4 centers and width of 1.

**Figure 12:**
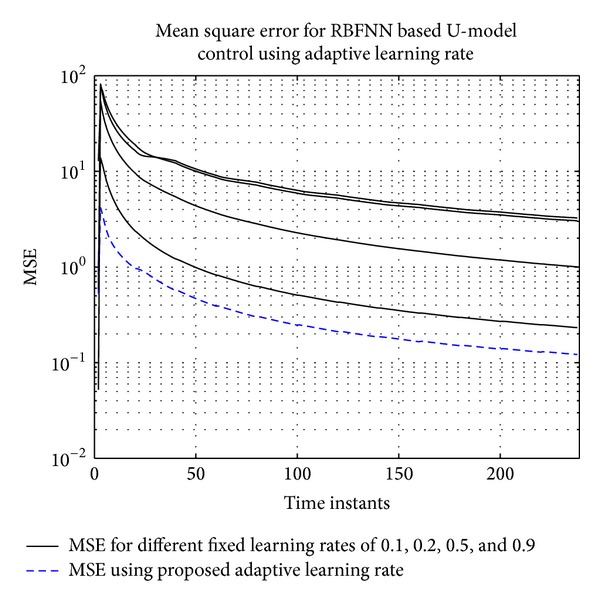
MSE for the tracking of 2-input 2-ouput system using RBFNN based U-model with fixed and adaptive learning rates. The fixed learning rates are 0.1, 0.2, 0.5, and 0.9.

For fixed learning rate case, learning rates of 0.1, 0.2, 0.5, and 0.9 are used. MSE for different learning rates and proposed adaptive learning rate are shown in Figure 12.

**Figure 13:**
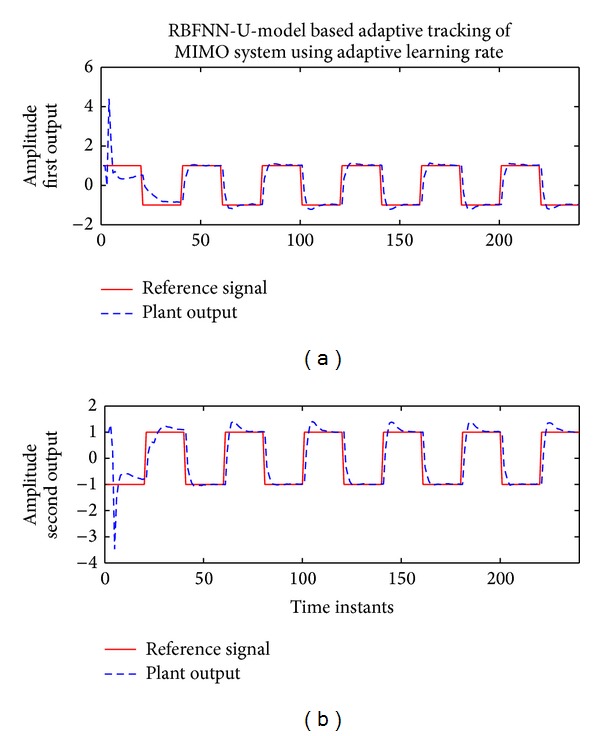
Tracking using RBFNN based U-model with Proposed Learning Rate Bound.

**Figure 14:**
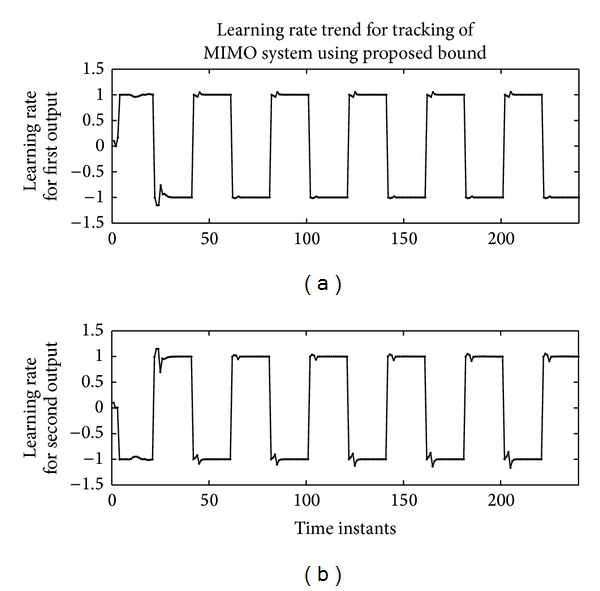
Learning rate trend for the tracking control of 2-input 2-output system using RBFNN based U-model.

It can be seen that adaptive learning rate has outperformed the fixed learning rate. The tracking of actual and identified nonminimum phase plant using the proposed approach is shown in Figure 13. The learning rate trend can be seen in Figure 14.

## 8. Conclusions

This paper presents the convergence analysis of the RBFNN with a deterministic framework. An adaptive learning rate is designed which is a result of time domain feedback analysis of RBFNN learning algorithm. The proposed adaptive rule for the learning rate gives faster convergence via an estimate of error energy while giving guarantee to the *l*_2_ stability governed by the upper bounding via small gain theorem. Performance of the proposed adaptive learning rate is verified by a number of identification and tracking control examples of nonlinear systems. The effectiveness of the proposed approach is observed by better MSE compared to the one with a fixed learning rate.
